# Astrocyte Regulation of CNS Inflammation and Remyelination

**DOI:** 10.3390/brainsci3031109

**Published:** 2013-07-22

**Authors:** Kumiko I. Claycomb, Kasey M. Johnson, Paige N. Winokur, Anthony V. Sacino, Stephen J. Crocker

**Affiliations:** Department of Neuroscience, University of Connecticut Health Center, 263 Farmington Avenue, Farmington, CT 06030, USA; E-Mails: ijichi@student.uchc.edu (K.I.C.); kasjohnson@student.uchc.edu (K.M.J.); gym215@sbcglobal.net (P.N.W.); sacino@hartford.edu (A.V.S.)

**Keywords:** astrocyte, heterogeneity, chemokine, antigen presentation, multiple sclerosis, leukodystrophy, transplantation

## Abstract

Astrocytes regulate fundamentally important functions to maintain central nervous system (CNS) homeostasis. Altered astrocytic function is now recognized as a primary contributing factor to an increasing number of neurological diseases. In this review, we provide an overview of our rapidly developing understanding of the basal and inflammatory functions of astrocytes as mediators of CNS responsiveness to inflammation and injury. Specifically, we elaborate on ways that astrocytes actively participate in the pathogenesis of demyelinating diseases of the CNS through their immunomodulatory roles as CNS antigen presenting cells, modulators of blood brain barrier function and as a source of chemokines and cytokines. We also outline how changes in the extracellular matrix can modulate astrocytes phenotypically, resulting in dysregulation of astrocytic responses during inflammatory injury. We also relate recent studies describing newly identified roles for astrocytes in leukodystrophies. Finally, we describe recent advances in how adapting this increasing breadth of knowledge on astrocytes has fostered new ways of thinking about human diseases, which offer potential to modulate astrocytic heterogeneity and plasticity towards therapeutic gain. In summary, recent studies have provided improved insight in a wide variety of neuroinflammatory and demyelinating diseases, and future research on astrocyte pathophysiology is expected to provide new perspectives on these diseases, for which new treatment modalities are increasingly necessary.

## 1. Introductions

Multiple sclerosis (MS) is the most common demyelinating disease of the CNS [1]. It is also the most common neurological disease among young adults affecting approximately 2.5 million people worldwide [2]. MS is a heterogeneous central nervous system (CNS) disorder associated with components of autoimmunity, genetic predisposition, and environmental factors. The clinical course of MS can vary between individuals, although periodic exacerbations of disability (relapses) punctuated by spontaneous improvements (remissions) represent the most prevalent form of this disease. It is important to note that while the immune system plays an important role in mediating the clinical exacerbations among patients with relapsing-remitting MS (RR-MS), disease progression is not modified by current immunomodulatory therapies and indicates that key aspects of disease pathogenesis are independent of autoimmunity [3]. In a recent clinical study, a comparison between RR-MS patients receiving interferon (IFN)-β immunomodulatory treatment and RR-MS patients not receiving this treatment were found to have the same progression of disability [3]. This finding indicates that our current understanding on the etiology of MS and its pathogenesis are incomplete and that dysfunction of non-hematopoietic cells, such as astrocytes, may play a more prominent role in the progression of MS than previously recognized [4,5,6]. 

Astrocytes have been long identified as reactive components within and surrounding demyelinated lesions in MS. Astrogliosis was one of the pathological hallmarks in the earliest descriptions of this disease pathology [7]; yet, until recently, the highly reactive state of astrocytes at white matter lesions was considered a secondary response to the primary immune response and followed demyelination. In this review, we outline how the role of astrocytes in MS is changing with recent advances in understanding of these cells. 

Astrocytes are the most numerous cell type in the CNS, which represents their potential to impact a wide range of homeostatic and pathological functions [8]. Astrocytes are typically identified by their expression of intermediate filament proteins, such as glial fibrillary acidic protein (GFAP) and vimentin [9]. Astrocytes exhibit significant morphological diversity, which also reflects inherent functional specialization among this population of cells. In the naive CNS, astrocytes can be broadly categorized into five distinct types based on their anatomical locations: (1) white matter astrocytes, which have numerous fine membranous projections giving them a star-like appearance; (2) gray matter astrocytes, which have a less complex shape and are often referred to as “protoplasmic”, (3) ependymal astrocytes, which are GFAP+ cells found within the stem cell niches of the brain that may be a form of progenitor cell; (4) radial glia found within the deep layers of the cerebral cortex that send projections toward the pial membrane and provide a scaffold for migrating neurons during brain development, and (5) perivascular astrocytes, which are GFAP+ cells with specialized projections called “vascular feet” that surround the neurovasculature. Increasingly recognized for their diverse functions in CNS homeostasis, astrocytes can serve many functions including acting as guards to CNS injury and inflammation.

Given the ubiquitous responsiveness of astrocytes to brain disease or trauma, in this review we will address the following questions; how do astrocytes contribute to immune-mediated demyelinating diseases? What determines the function of astrogliosis? And, how can our current answers to these questions be adapted toward increasing our understanding of MS pathology for therapeutic benefit to treat MS? 

## 2. Astrocytic Regulation of Adaptive Immune Responses

There are many hypotheses on the initiation and development of autoimmunity in MS, in which the erroneous recognition of self-antigens may result from pathogen infections [10] or an *a priori* neurodegenerative condition [5,6]. Given the prominent association of T-cell mediated immunity with MS, there are several plausible means by which astrocytes could foster autoimmunity. First, astrocytes may facilitate immune cell extravasation into the CNS by releasing chemoattractant cytokines (*i.e.*, chemokines). Secondly, they may promote autoimmunity through antigen presentation. And lastly, astrocytes enhance T cell activation through modulating the activity of innate immune cells (*i.e.*, microglia). When considering the potential for astrocytes to inflammation within the CNS during MS, the pathogenic potential of these actions may also represent potential mechanisms by which astrocytes could also dampen inflammation to promote remyelination.

One critical function of astrocytes is acting as sentinels and monitoring of the neuro-vasculature called the blood-brain barrier (BBB). This specialized multi-cellular unit is organized in a multi-laminal structure of the blood vessels within in the CNS affording strict regulation of trafficking of water, ions, nutrients, and cells from peripheral circulation into and out of the brain parenchyma. The BBB offers an anatomical mechanism for highly selective passage into the CNS compartment and astrocytes play an important function in modulating its function. This selectivity was historically considered to foster an immunoprivileged environment for the brain, although the notion has been revised as immune cells and immune surveillance of the CNS is now recognized as a requirement for health and homeostasis [11]. It is well established that under inflammatory conditions, the integrity and function of the BBB is modified and enables greater leukocyte passage into the CNS. Using loss of function studies, Voskuhl *et al*. [12] showed that astrocytes play a critical role in shielding the CNS during inflammatory responses through their actions at the perivascular interface. Another study by Owens and colleagues also indicate that astrocyte ablation results in enhanced inflammatory monocyte cell migration into the CNS [13]. Interestingly, another cell type tightly associated with BBB physiology is the pericyte [14,15]; however, the functional interaction between pericytes and astrocytes in the context of CNS inflammation is an area of active study. 

The extent of astrocytic diversity described above has broad implications in many roles. For instance, astrocytes secrete co-stimulatory factors for T cell activation, which can play a regulatory role in reactivation of autoreactive immune cells as they enter the CNS [16]. It has also been reported that subpopulations of astrocytes have phagocytic activity that represents their immunomodulatory function as *de facto* antigen presenting cells (APCs) [17]. In this capacity, astrocytes could foster adaptive immune responses and ultimately exacerbate autoimmune diseases of the CNS, such as MS. As the most abundant glial cell type exposed to early T cell infiltration, astrocytes likely serve immune-related purposes. In addition to their ability to express major histocompatibility class II (MHC II) antigens in murine model and human MS upon IFN-γ stimulation [17,18], preliminary evidence of CNS cells as effective antigen presenters arose when myelin-specific T cells localized to and remained within the CNS following activation *in vitro* [19]. Astrocytes also express CD80 and CD86, cell surface proteins potently associated with T cell activation, and blockade of these proteins hampers T cell activation [20]. Also, when astrocytes are exposed to interferon-gamma (IFN-γ), a pro-inflammatory cytokine made by the T cells, they can enhance the proliferation rate of myelin oligodendrocyte glycoprotein (MOG)- and proteolipid protein (PLP)-specific T cells [21,22]. These findings are consistent with previous finding that activated astrocytes upregulate intercellular adhesion molecule-1 (ICAM-1) and vascular cell adhesion molecule-1 (VCAM-1) that promote cell-cell interactions with surrounding leukocytes [23]. Together these findings indicate that astrocytes contain the cellular machinery necessary to deliver signals required for T cell activation and may support a pro-inflammatory function for astrocytes in T cell mediated CNS injury. 

Another possible way that astrocytes may promote T cell mediated CNS injury is to serve as antigen presenting cells (APCs). One plausible mechanism by which antigen presentation by astrocytes may contribute to pathology in MS is the β_2_ adrenergic receptor. Functionally, these receptors constitutively suppress MHC II expression by increasing intracellular cAMP levels through PKA activation [24]. Once activated, PKA phosphorylates the MHC II transactivator (CIITA), which in turn inhibits MHC II transcriptionally, thereby regulating global antigen presentation activity. This regulatory pathway of APC function has also been analyzed in EAE models [25]. Importantly, astrocytes in white matter lesions in MS patients have also been reported to express significantly lower level of β_2_ adrenergic receptors suggesting potential for greater APC activity [26]. Co-factors for MHC II function, including CD80, CD86, and CD40, which are critical for T cell receptor (TCR) binding, can also be expressed by astrocytes [17,23,27]. Like better known “professional APCs”, including macrophages and dendritic cells (DCs) that constitutively express MHC II molecules, astrocytes also express MHC II [17]. Cytokines shown to be expressed during immune-mediated myelin injury, including IFN-γ and tumor necrosis factor-alpha (TNF-α), have been reported to induce an upregulation of MHC II genes in astrocytes [23,28,29]. Thus, within the inflammatory milieu of the MS brain, and as modeled in mice by EAE, astrocytes are capable of expressing all of the essential subunits required for antigen presentation functions [30]. 

Despite compelling experimental *in vitro* and *in vivo* findings, the contribution of astrocytic APC functions toward autoreactivity in MS remains controversial. Even if astrocytes do not present antigen directly, they undoubtedly expedite the process by secreting chemokines that attract DCs to damaged myelin [31]. For instance, Hassan-Zahraee *et al*. [32] explored the potential for human astrocyte cultures to generate non-specific inflammatory responses to “superantigens”, which result in T cell activation. Another facilitatory role of astrocytes is that they may serve as secondary APCs based on their ability to efficiently present epitopes of MOG but not the full-length MOG protein [22]. In the latter scenario, unlike conventional APCs that promote Th1 phenotype in T cells, astrocytic APC functions promote mixed Th1 and Th2 T cell phenotypes. Thus, astrocytic APC functions may modulate the overall inflammatory environment of the CNS in MS by promoting a Th2 repertoire of cytokines. The contribution of astrocytes toward specific regulation of additional T cell subtypes in MS, such as regulatory T cells, is less clear. However, not all reported actions of astrocytes are to facilitate autoreactivity; a recent study by Schlüter and colleagues suggest a potentially important role for astrocytic expression of Fas, an inducer of programmed cell death, in the activation of autoreactive T cell apoptosis [33]. Thus, astrocytes may also limit T cell survival within the CNS. Collectively, these data indicate that astrocytes play potentially central roles by serving as professional APCs within the CNS and thereby act to regulate autoreactive T cell functions.

Accumulating evidence also indicates that astrocyte functions may underlie the susceptibility to chronic infections. This concept is best illustrated by studies exploring the cellular basis for strain difference susceptibility to Theiler’s murine encephalomyelitis virus (TMEV)-induced demyelination. For instance, bone marrow chimera studies between susceptible and resistant strains of mice have shown that strain susceptibility to infection and demyelination is accountable to cells of non-hematopoietic origin [34]. To address the involvement of non-hematopoietic stem cells within the brain that have immunocompetence, Carpentier *et al.* [35] proposed astrocytic regulation of virally-induced CNS infection as a factor in strain susceptibility of TMEV-induced demyelinating disease (TMEV-IDD). A current hypothesis suggests that differential astrocytic expression of cytokines, chemokines, and adhesion molecules underlies the susceptibility of mouse strains to TMEV since both susceptible and resistant mouse strains mount efficient antiviral T cell responses [35]. The nature of this complex, astrocyte-regulated immunity requires further research to expand our current understanding how these differences manage the differential susceptibility to CNS virus infections related to demyelinating diseases.

## 3. Astrocytic Regulation of Innate Immune Responses

Regulation of innate immunity in the CNS may represent a fundamental role of astrocytes to maintain homeostasis. Recent studies indicate a prerequisite for innate immune responses (*i.e.*, microglia) in the development of adaptive autoreactive T cell-mediated demyelination [36,37]. Microglia represent only 5%–20% of the CNS cell population but are responsible for CNS surveillance for detection of foreign pathogens [38]. For example, microglial activation is tightly regulated via multiple mechanisms. One such, mechanism involves the cell-surface protein-protein interaction between CD200 on astrocytes and its cognate receptor CD200R expressed by microglia [39,40]. Disruption of this physical interaction between astrocytes and microglia, results in a loss of constitutive inhibition and in activation of the microglial cells [39]. Numerous studies now support a prominent role for microglia on disease progression and myelin pathology [41,42,43]. It was reported that astrocytes physically communicate with microglia and oligodendrocytes through several connexins. For instance, dysfunction of connexin 43/47 and connexin 30/32 in astrocytic gap junctions has been implicated to play a role in pathogenesis in several demyelinating disease models [44,45,46]. Also, indirect mode of interactions between astrocytes and oligodendrocytes, specifically through secreted molecules, has been reported [47,48].

In addition to many findings of microglia activating astrocytes, microglial activation by astrocytes has been evident in multiple studies. For instance, Davalos and colleagues reported that activated astrocytes in response to focal traumatic insults in the CNS releases adenosine 5′-triphosphate (ATP), resulting in immediate activation of microglia through purinergic receptors both morphologically and functionally [49]. Activated microglia then secrete many immunomodulatory factors, such as interleukin (IL)-1β, IL-6, plasminogen, and TNF-α [50,51,52,53]. In other CNS disease models, such as neonatal Borna disease virus infection, astrocytic activation proceeds and contributes to microglial activation *in vitro* through an unknown secreted protein, which is heat-resistant and has a low molecular weight [54]. In RR-MS case where they develop permanent and progressive disability (secondary progressive MS), astrocytic chemokines, including CCL2 and CXCL10 that are predominantly produced by reactive astrocytes, may be a major activator of microglia at the rim of the plaque and demyelinating sites [55].

Also, it has been suggested that astrocytes modulate microglial activation. For instance, microglia exposed to pro-inflammatory stimuli, such as IL-1β, are activated and increase production of reactive oxygen species. Interestingly, using an *in vitro* system, isolated microglia pre-treated with astrocyte-conditioned media prior to challenge with hydrogen peroxide increased the production of antioxidants, indicative of a counteracting response to microglial activation [56]. Together, maintaining the intricate balance of activation/modulation between astrocytes and microglia may be critical in determining the predominance of processes mediating either disease or tissue repair.

## 4. Regulation of Astrocytic Phenotype Influences Oligodendrocyte Differentiation

What factors are most important in determining the phenotype of astrocytes? One possibility that has garnered increasing attention from a series of recent reports is the extracellular matrix (ECM). The ECM is well known to influence the behavior of many cell types, and the potent impact of ECM proteins is now highlighted as modifiers of astrocyte function. *In vitro* studies have shown that astrocytes behave and function differently depending upon the ECM protein on which they are grown. For instance, Barnett and colleagues recently explored the idea that astrocyte activation is an ECM substrate-dependent phenomenon [48]. There are many common ECM proteins, including laminin, vitronectin, fibronectin (Fn), and tenascin-c (TnC). Nash *et al*. examined whether astrocytes grown on different ECM proteins would have differing activity levels, as indicated by immunoreactivity of GFAP, but also have an effect on astrocytic gene expression [48]. Specifically, they found that astrocytes grown on TnC exhibited a quiescent phenotype and were unable to support myelination in culture. In contrast, astrocytes grown on poly-l-lysine (PLL), a standard culture coating protein, were able to support myelination and exhibited an active phenotype [48]. Microarray analysis of gene expression determined that TnC evoked differential gene expression from the PLL astrocytes, with significantly more CXCL10 chemokine expressed by astrocytes grown on TnC than their PLL counterparts [48]. These data suggests that CXCL10 may prevent the myelination in culture [48]. Thus, not only does the ECM impact the overt appearance of astrocytes, but it also has the potential to alter the immunomodulatory function of astrocytes [57] and their propensity to support remyelination [48].

Coincidently, astrocytes are prominent producers of ECM molecules that can impact oligodendrocyte maturation [58]. With reference to TnC as a potent inhibitory ECM substrate for astrocytes and oligodendrocyte progenitor cells (OPCs), astrocytes are also recognized as a source of TnC in chronic myelin lesions in MS [59]. In the acute setting, however, the production of Fn by astrocytes has also been demonstrated to have a negative impact of OPC differentiation. Baron and colleagues have very recently reported that astrocytic expression of Fn is induced robustly in models of immune-mediated myelin injury and its expression by astrocytes can be upregulated in culture in response to inflammatory stimulation (e.g., lipopolysaccharide) [60]. Astrocytic Fn, while not directly toxic to OPCs or oligodendrocytes, was reported to markedly attenuate OPC differentiation *in vitro*. When astrocytic Fn aggregates were injected into experimental white matter lesions induced by lysolecithin, remyelination was impaired [60]. The idea that ECM modifies the functional phenotype of astrocytes proposes an interesting model to better understand how the complex milieu of myelin lesions in MS affects the role of astrocytes in this disease. These findings also implicate intergrins, ECM receptors, as plausible targets for modulating the negative effects of ECM proteins on specific cell types.

It is also worth noting that in addition to ECM proteins, astrocytes secrete a myriad of factors that can have wide-ranging effects on OPC differentiation and remyelination [61]. Other examples of astrocyte-derived proteins associated with the extracellular matrix that can have a dramatic effect on impairing remyelination include high molecular weight hyaluronan (HA) [62]. Accumulation of HA in inflammatory lesions in both MS and mouse models has been implicated in remyelination failure. Interestingly, CD44, an HA receptor, is expressed on “reactive” astrocytes, which has been associated with loss of cell-cell contact and implicated in neuropathology [63]. Thus, astrocytic dysfunction may contribute to the consequences of inflammatory HA accumulation in white matter pathology. Moreover, these studies point to an interesting role for modifications in the local environment of myelin lesions as important regulators of astrocytic potential and astrocyte function(s). It is now accepted that what we refer to as “reactive” astrocytes is not a consequence of a single factor produced only when astrocytes are activated. Indeed, the profile of the many factors produced by reactive astrocytes is uniquely different from resting, protoplasmic astrocytes [64]. Understanding these profile changes, in combination with an understanding of how the ECM regulates these differences, would be expected to provide additional bases for interrogating the functional impact of astrocytes in specific disease states.

## 5. Phenotypic Plasticity of Astrocytes as Regulated by Inflammation

As described earlier, astrocytes undergo a spectrum of shapes, which can reflect their functional phenotypes; quiescent, as seen in the normal uninjured CNS, to reactive, as seen after injury or disease. The activity level is associated with cellular hypertrophy, proliferation, process extension, and increased production of GFAP, vimentin, nestin, heparan sulfate proteoglycans, chondroitin sulfate proteoglycans, and growth factors [65,66,67,68,69]. One question regarding the generalized function of reactive gliosis is that of functional heterogeneity. Given its prominent role in a variety of neurodegenerative conditions, and its pleiotropic activation of astrocytes, the response to the pro-inflammatory cytokine IL-1β has become a practical model for study of astrocytic behaviors [27].

IL-1β is an important component for the initiation of an astrocytic response to injury and for the formation of astrocytic scar after CNS injury. For instance, IL-1β has been shown to up-regulate certain genes associated with neuronal and glial growth and survival, such as IL-6, ciliary neurotrophic factor, and nerve growth factor in primary astrocyte cultures [70]. Other up-regulated genes are those from the TNF superfamily, which activate signaling pathways involved in cell survival, death, differentiation, development, organization and homeostasis. IL-1β also induces the expression of members of the chemokine family, or chemoattractant cytokines, including the CXC, CX3C, and CC subfamilies. The astrocytes within the glial scar undergo a dramatic change in phenotype resulting in a reactive phenotype described by up-regulation of GFAP, vimentin, and hypertrophy; however, the mechanism by which IL-1β evokes this reactive phenotype has remained elusive. One intracellular pathway, the Rho GTPase-Rho kinase (ROCK) pathway, has been shown to mediate some of the astrocytic phenotype in response to IL-1β. This well studied pathway has been shown to be involved in modulating changes in cellular morphology and cellular migration through F-actin and its interactions with focal adhesions, non-muscle myosin, and microvillar adapter proteins of the ezrin-radixin-moesin (ERM) family [27]. Also, microglial IL-1β is reported to activate astrocytes through a connexin 43-dependant gap junction between astrocytes [71,72], which also relates these effects to gliotransmission [8].

Important questions stemming from these findings include what factors contribute to the control astrocyte activation? And, what influences the functional phenotype of astrocytes to perform beneficial or detrimental functions? One candidate protein we have been studying that has a potentially important regulating activity on astrocyte function is tissue inhibitor of metalloproteinase-1 (TIMP-1). This extracellular dual function protein is predominantly expressed by astrocytes in the CNS in response to stress, injury or inflammation [73]. Induction of TIMP-1 expression from astrocytes is not a ubiquitous response to a broad range of stimuli, rather, robust and regulated TIMP-1 expression can be observed in response to a limited number of factors that include IL-1β [74,75,76]. We have shown that TIMP-1 is itself a potent astrocyte mitogen [47]. In EAE, astrocytic expression of TIMP-1 is spatially associated with white matter lesions [77], and we have determined that TIMP-1 deficient mice exhibit delayed developmental myelination and failure to remyelinate when myelin is injured as adults [47,78]. Importantly, expression of TIMP-1 by astrocytes is labile; it is robustly induced by IL-1β but then repressed with “chronic” exposure to this cytokine [74,79]. Coincidently, analysis of TIMP-1 expression in acute demyelinating diseases that also show remyelination has shown robust expression of TIMP-1 [80], whereas cerebrospinal levels of TIMP-1 across multiple clinical phenotypes of MS indicate no induction of TIMP-1 in this chronic inflammatory disease [81]. These clinical data provide an interesting correlate with animal studies showing a positive correlation between TIMP-1 levels and the propensity for remyelination in the CNS. Study into the dysregulation of astrocytic TIMP-1 expression and how its secretion by astrocytes modulates their function in white matter repair is ongoing.

## 6. Astrocyte Dysfunction in Leukodystrophies

An emerging trend in our understanding of neurodegenerative diseases of the CNS is the increasing prominence of the role of astrocytes, no longer a passive support network for neurons, the abundant population of astrocytes in the CNS has been documented to have a primary role in a variety of neurological conditions. This topic has been well described in a very recent review [82]. Thus, we elaborate on the overall notion that dysfunction of astrocytes underlies development of CNS inflammation [83]. The development of pathological astrocytic functions, whether through loss of essential basal trophic actions or gain of specific toxic functions, leads to a condition we refer to as “*gliodystrophy*” [84]. Indeed, the primary role of astrocytes in an increasing number of degenerative CNS diseases has prompted a revised nomenclature for these types of neurological conditions, gliopathy [82]. In the following section, we turn our attention to the role of astrocytes in a class of demyelinating diseases called leukodystrophies; a group of predominantly fatal demyelinating diseases with genetic causes. Specifically, we focus on a newly described role of astrocytes in a rare but fatal leukodystrophy, called Krabbe disease.

Krabbe disease, also known as globoid cell leukodystrophy (GLD) is a well-characterized genetic demyelinating disease, caused by genetic mutations in a lysosomal enzyme, resulting in accumulation of a cytotoxic lipid, psychosine. It has been hypothesized that the toxicity of supraphysiologic level of psychosine kills oligodendrocytes, resulting in severe demyelination. However, when oligodendrocytes from *twitcher* mice, a murine model of GLD, were transplanted to *shiverer* mice, another mouse model for demyelination, the twitcher oligodendrocytes were capable of myelinating the shiverer axons [85]. This suggests that demyelination in GLD is not exclusively attributable to oligodendrocytes dying from lipid accumulation, based on its capability to myelinate axons in different cellular environment. 

**Figure 1:**
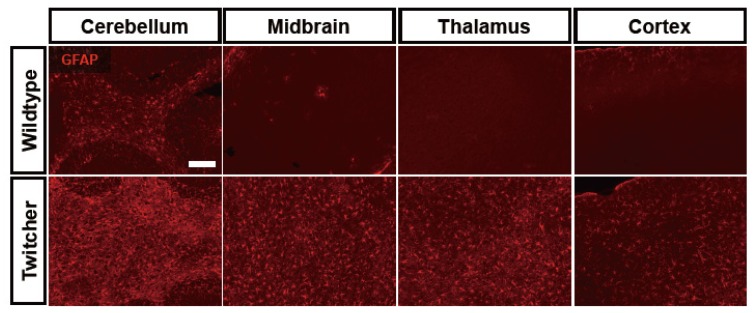
Widespread astrogliosis is a prominent neuropathological feature of the twitcher mouse brain. Representative pictures show immunohistochemically labeled glial fibrillary acidic protein (GFAP)+ astrocytes (red) in several regions of the brain, including cerebellum, midbrain, thalamus, and cortex in wildtype C57BL/6 (upper row) and in age-matched twitcher mice (bottom row). Note that highly fibrous GFAP immunoreactivity is observed with greater intensity throughout the twitcher mouse brain, compared to wildtype mouse. Scare bar = 250 μm.

We have recently implicated astrocytes in the pathogenesis of neuropathology in GLD. We determined that astrocytic expression of matrix metalloproteinase (MMP)-3, an extracellular protease, is dramatically increased at the time of clinical disease onset in the twitcher mice. Furthermore, its expression continues to elevate with disease progression [86]. This astrocytic MMP-3, which potentially targets myelin protein proteolytically, is a primary mediator of the formation of multinucleated globoid cells, highly activated phagocytes and a hallmark of GLD pathology [86]. In addition, it was reported that the production of hematopoietic prostaglandin synthase (HPGDS) and PGD2 in microglia is significantly increased along with elevated expression of astrocytic PGD2 receptors, DP1 and DP2 [87]. Pharmacological blockade of HPGDS or genetic ablation of DP1 in the twitcher mouse resulted in decreased astrogliosis and microgliosis, accompanying by less demyelination [87]. Thus, astrocytic reactivity in the CNS of GLD may not represent a secondary response to demyelination, but rather may be a primary response to accumulated psychosine in this disease that may contribute significantly to the pathogenesis of GLD (Figure 1). Further study on the regulation of astrocyte reactivity in this disease may represent a new avenue for understanding the etiology of neuropathology in GLD.

Another example of astrocyte involvement in a leukodystrophy is shown in vanishing white matter disease (VWM), also known as childhood ataxia with diffuse CNS hypomyelination [88,89]. Early neuroimaging studies examining the cortex of patients with VWM identified significant reduction in white matter [90]. Genetic mutations identified in the gene encoding translation initiation factor 2B (eIF2B) have been attributed to the cause of this disease [91,92]. Interestingly, neither neuronal nor oligodendrocyte cultures from VWM patients exhibited any remarkable abnormalities in proliferation, differentiation or maturation, when compared to cells derived from non-disease cases [93]. In contrast, patient-derived astrocytes from VWM were fewer in number and exhibited greatly reduced proliferation rates even when stimulated with bone morphogenic protein 4 (BMP4) when compared to control human astrocyte cultures [93]. In fact, reactive astrocytes in the cortex of VWM patients are not as fibrous as those in healthy control [94]. These findings suggest that a key pathological difference in the CNS of VWM is associated with a deficiency of astrocytic support during early development or homeostasis in postnatal maturation. To date, the specific mechanism underlying this hypotrophy of astrocytes in VWM and their putative contribution to the pathogenesis of VWM has yet to be determined.

These examples suggest that astrocytes are considerably involved in the demyelinating process of leukodystrophies. The specific nature of astrocytic participation in each condition given the disparity of genetic causes, clinical courses and altered metabolism, would suggest that the role(s) of astrocytes likely differs in each demyelinating condition. Moreover, in contradiction with our discussion on the role of astrocytes in adaptive immunity, the non-T cell mediated nature of myelin perturbation in leukodystrophies may also indicate that astroglial changes fundamentally differ from their putative roles in myelin destruction or repair process in RR-MS in which adaptive immune response appears to drive the primary process. On the other hand, primary-progressive form of MS (PPMS), in which immuno-modulatory treatments are not effective, unlike RR-MS patients, resembles leukodystrophies in the context of the pattern of disease course and pathological findings, including the involvement of CNS innate immune system and astrocytic abnormality. This suggests that the pathogenic mechanism of PPMS and leukodystrophies may greatly overlap, despite the fact that these diseases are known to be entirely different entities. Understanding astrocytic contribution to each disease process, either positively or negatively, may reveal a full range of astrocytic functions in the CNS.

## 7. Conclusions

The manifold functions of astrocytes has prompted increased awareness on the potential of these cells both to maintain CNS homeostasis and their contribution(s) to CNS pathology. Herein, we have outlined current data and understanding on the roles and actions of astrocytes related to demyelinating diseases of the CNS that include inflammatory modulation, antigen presentation, and gliotransmission. A central theme of this field is the awareness that astrocytic responses are intimately linked to changes in their environment that then contribute to astrocyte heterogeneity both in form and function. We provided examples from studies on MS, and recent work on leukodystrophies, which have revealed that astrocytes contribute significantly to the pathology of demyelinating diseases. Therapeutic adaptation of astrocyte plasticity may hold promise to foster remyelination in neurodegenerative diseases by coaxing astrocytic functions toward repair and homeostasis. 

## 8. Therapeutic Potential of Astrocytes

Is astrocytic inflammation sufficient? We and others have previously described how factors secreted by astrocytes have the potential to either promote or impair the differentiation of oligodendrocytes which may either foster remyelination or its failure in demyelinating disease like MS. In a series of seminal studies, Campbell and others have demonstrated the pathological potential of astrocyte derived cytokines and chemokines [95]. For example, by adopting astrocyte-specific (GFAP promoter-driven) transgenic expression of IL-17A, IL-6, IL-12 or CXCL10 they have demonstrated that astrocytic production of these potent inflammatory factors are sufficient to elicit glial activation [96,97]. While in many cases the singular production of these factors can result in mild neuroinflammation with recruitment of peripheral immune cells, these changes set the stage for profound neuropathology in response to a variety of infectious, inflammatory or injurious treatments [98,99,100]. Thus, these studies lend further support to the notion that early initiating steps of pathology within the MS brain, perhaps by astrocytes, may precede the pathogenic development of peripheral adaptive immune response in this disease. Moreover, cytokines and chemokines represent only a small sampling of the full repertoire of extracellular proteins made by astrocytes [101,102]. Ongoing studies to further interrogate the astrocyte secretome to determine how it is modified by disease, genetic mutations and/or inflammation would be expected to provide important insights into the range of biological roles astrocytes play in neurodegenerative illnesses.

How can we harness our current knowledge to provide therapeutic benefit for individuals with neurological diseases? One avenue of intensive investigation is the notion of astrocyte transplantation. Compelling findings from Proschel and others have eloquently demonstrated that *a priori* modification of astrocyte phenotype has a marked effect on the success and outcome of astrocytes transplanted into spinal contusion injury models. One of their studies demonstrated that exposure of human glial progenitor cells to BMP prior to transplantation into rats with focal spinal transection injuries promoted neurite outgrowth and recovery of locomotor functions [103]. In essence, these results demonstrate the potential utility of exploiting astrocytic heterogeneity for therapeutic gain [104]. On potential drawback to such a transplant approach for how one might view treating MS is the random, widespread, unpredictable and recurrent nature of myelin lesions. Nevertheless, knowledge gleaned from these types of studies would be expected to elucidate fundamental differences between positive and negative astrocytic phenotypes, which could then be targeted by other, possibly pharmacological means.

Importantly, more rigorous study of human astrocytes may be warranted as it has long been recognized that human astrocytes are larger and more complex than that of the *rattus* or *mus* genera. Recently, Han *et al*. [105] reported that human astrocytes when transplanted into the early postnatal CNS of mice integrate into the host nervous tissues and resulted in a dramatic improvement in cognitive function. When considered in the context of the discussion of this review, one might consider the potential of this approach to explore how disease-specific induced pluripotent stem cell (iPSC)-derived astrocytes when transplanted into naive recipient mice might impact the pathological outcomes among widely used models of neurodegenerative and demyelinating diseases. For instance, it is known that astrocytes derived from amyotrophic lateral sclerosis (ALS) cases, when transplanted into naive rodents, elicit neurodegenerative changes that recapitulate many aspects of this dreadful disease [106]. While the astrocytic-source of neurotoxicity for ALS is well recognized, the potential role for astrocyte-driven pathology among more enigmatic diseases, including Parkinson’s disease or MS, has not been explored in this way. Future studies examining the disease modifying potential of human astrocytes in murine models may provide interesting new insights into whether disease-specific astrocytes influence disease processes modeled *in vivo*. Findings from the many lines of investigation mentioned in this review are expected to advance our understanding on the contribution of astrocytes to the pathogenesis of myelin injury and repair in diseases like MS. It is expected that accumulating evidence supporting fundamental roles for astrocytes in neurodegenerative diseases will also lead to an increased need for therapeutic targeting of astrocytes that may address an ever-increasing need for new treatments for diseases.
